# Detection of Chlamydial DNA from Mediterranean Loggerhead Sea Turtles in Southern Italy

**DOI:** 10.3390/ani12060715

**Published:** 2022-03-11

**Authors:** Antonino Pace, Nadia Vicari, Sara Rigamonti, Simone Magnino, Luca Borrelli, Ludovico Dipineto, Alessandro Fioretti, Sandra Hochscheid, Luís Tavares, Ana Duarte

**Affiliations:** 1 Marine Turtle Research Group, Department of Marine Animal Conservation and Public Engagement, Stazione Zoologica Anton Dohrn, 80055 Portici, Italy; sandra.hochscheid@szn.it; 2 Department of Veterinary Medicine and Animal Productions, Università degli Studi di Napoli Federico II, 80137 Naples, Italy; luca.borrelli@unina.it (L.B.); ludovico.dipineto@unina.it (L.D.); fioretti@unina.it (A.F.); 3 National Reference Laboratory for Animal Chlamydioses, Istituto Zooprofilattico Sperimentale della Lombardia e dell’Emilia Romagna “Bruno Ubertini”, Sede Territoriale di Pavia, 27100 Pavia, Italy; nadia.vicari@izsler.it (N.V.); sara.rigamonti@izsler.it (S.R.); simma62@gmail.com (S.M.); 4 Centro de Investigação Interdisciplinar em Sanidade Animal, Faculdade de Medicina Veterinária, Universidade de Lisboa, 1300-477 Lisbon, Portugal; ltavares@fmv.ulisboa.pt (L.T.); anaduarte@fmv.ulisboa.pt (A.D.)

**Keywords:** *Caretta caretta*, *Chlamydia* spp., *C. psittaci*, *C. pneumoniae*, chlamydia-like organisms, molecular diagnosis, zoonosis, Mediterranean Sea

## Abstract

**Simple Summary:**

Chlamydiae are ubiquitous in animals, particularly in wildlife. Some chlamydial species additionally represent a potential risk for public health, as they have been associated with severe diseases in humans. Chlamydial agents have been detected in several groups of reptiles, but these animals do not always show signs of disease. Therefore, the present study aimed at investigating the presence of chlamydial DNA in samples collected from asymptomatic Mediterranean loggerhead sea turtles, after rehabilitation in a research centre in southern Italy. The molecular analyses resulted in the extensive presence of chlamydial DNA in the examined samples, suggesting that sea turtles might host these microorganisms as opportunistic flora, and potentially disseminate them. Despite the impossibility to identify the chlamydial species involved, this study emphasizes the importance of chlamydiae in sea turtles and motivates further studies to fully understand these agents, especially in relation to wildlife conservation and potential impacts on animal and public health.

**Abstract:**

Chlamydiae are obligate intracellular bacteria that include pathogens of human and veterinary importance. Several reptiles were reported to host chlamydial agents, but pathogenicity in these animals still needs clarification. Given that only one report of chlamydiosis was described in sea turtles, and that chlamydiae might also be detected in hosts without clinical signs, the current study examined asymptomatic Mediterranean loggerhead sea turtles for the presence of chlamydial DNA. Twenty loggerhead sea turtles, rehabilitated at the Marine Turtle Research Centre (Portici, Italy), were examined collecting ocular-conjunctival, oropharyngeal and nasal swabs. Samples were processed through quantitative and conventional PCR analyses to identify Chlamydiales and *Chlamydiaceae*, with particular attention to *C. pecorum*, *C. pneumoniae*, *C. psittaci*, and *C. trachomatis*. Although it was not possible to determine the species of chlamydiae involved, the detection of chlamydial DNA from the collected samples suggests that these microorganisms might act as opportunistic pathogens, and underlines the role of sea turtles as potential carriers. This study highlights the presence of chlamydial agents in sea turtles, and encourages further research to fully characterize these microorganisms, in order to improve the management of the health and conservation of these endangered species, and prevent potential zoonotic implications.

## 1. Introduction

The order Chlamydiales is composed of obligate intracellular bacteria, characterized by a distinctive biphasic developmental cycle, which involves two forms: an extracellular survival form (i.e., elementary body) and an intracellular replicating form (i.e., reticulate body) [1,2]. Chlamydial taxonomy has been subjected to several changes over the last few years, with the proposition of new candidate species and the inclusion of a wide range of Chlamydia-like organisms [3,4,5,6,7,8]. To date, nine families have been described, among which the *Chlamydiaceae* are probably the most extensively investigated, as they include important pathogens for human and animal health [2,9,10]. Indeed, three species in this family (i.e., *Chlamydia pneumoniae*, *C. psittaci* and *C. trachomatis*) have long been considered major human pathogens, responsible for a wide range of disorders in various systems (respiratory, gastrointestinal, nervous, musculoskeletal and reproductive) [9,11,12,13]. These species are no longer considered restricted to humans, since they have been detected in many vertebrates, where they might cause from asymptomatic to severe diseases [2,9,10,12,14,15].

Reptiles are being increasingly recognized as hosts to chlamydiae and their role as possible carriers has been reconsidered [9,10,16,17]. Since the first record in the eastern fence lizard (*Sceloporus undulatus*) over 70 years ago [18], chlamydial species have been later discovered in all major groups of reptiles (e.g., crocodiles, chameleons, iguanas, snakes, turtles, tortoises) [19,20,21,22,23]. In these animals, clinical manifestations might range from non-specific symptoms (e.g., lethargy, anorexia) to ocular disorders, respiratory infections, gastrointestinal lesions and granulomatous inflammation in multiple organs [2,10,16,19]. However, the pathogenic potential of chlamydial agents in reptilian hosts remains to be elucidated, given that these microorganisms have been detected from captive and free-ranging reptiles, with and without clinical signs [2,5,16]. Several authors have suggested that chlamydiae might act as commensal flora or conditional pathogens in reptiles, triggering the manifestation of disease when animals are exposed to other stressors (e.g., capture, transportation, temperature changes, malnutrition, overcrowding, co-infections, etc.) [2,21,24].

The same consideration applies to sea turtles, in which many bacteria, generally considered part of the environment or the turtles’ normal flora, might exhibit an opportunistic behaviour and express their pathogenic potential when the turtles’ immune response is compromised due to stressful conditions [25,26,27,28,29]. Therefore, any stress (poor water quality, injuries, captivity, etc.) could promote the takeover of opportunistic agents, as well as the manifestation of sub-clinical diseases or the reactivation of latent infections. This is probably the reason why infectious diseases have been often described among captive sea turtles [30,31,32]. To date, there has been only one report of chlamydiosis in sea turtles, which caused the death of hundreds of juvenile green turtles (*Chelonia mydas*) in a turtle farm [33]. Although the affected turtles showed non-specific symptoms (lethargy, debilitation and inability to dive), the disease appeared as a systemic infection, with necrotic lesions in many internal organs, mainly heart, spleen and liver. Subsequent analysis of tissue samples resulted in the detection of chlamydial agents in the macrophages (i.e., *Neochlamydia* spp., *C. abortus* and *C. pneumoniae*) [9,15,33].

Chlamydiosis diagnosis has always been challenging. Since culturing reptile chlamydiae is limited by technical constraints and low sensitivity, electron microscopy and immunohistochemistry have usually been recommended for reliability and rapidity [9,20,33]. However, nucleic acid amplification represents nowadays a powerful tool to detect chlamydial agents due to sensitivity, specificity and the possibility to better characterize the strain, by targeting specific sequences [2,5,34,35].

The literature on chlamydial infections in sea turtles is scarce, but these microorganisms might be important in clinically healthy animals. In light of that, the current study examined asymptomatic Mediterranean loggerhead sea turtles (*Caretta caretta*), utilizing molecular diagnostic techniques to detect the presence of *Chlamydiaceae*, focusing on *C. pecorum*, *C. pneumoniae*, *C. psittaci* and *C. trachomatis*.

## 2. Materials and Methods

### 2.1. Sampling

Samples were collected during May and June 2017 from a total of 20 loggerhead sea turtles, temporarily housed at the marine turtle rescue and rehabilitation centre (Stazione Zoologica Anton Dohrn of Naples, Naples, Italy). The majority of sea turtles were juveniles (mean curved carapace length 52.56 ± 3.66 cm; mean weight 21.73 ± 4.36 kg), with the exception of three mature adults. All sea turtles were recovered in near shore environments along the coasts of southern Italy, and were recruited in the present study following complete rehabilitation, after the responsible veterinarian declared the animals to be healthy and ready to be reintroduced in nature.

Sterile, cotton-tipped swabs were used to collect one oropharyngeal swab (labelled a), one ocular-conjunctival swab (labelled b) and one nasal swab (labelled c) from each animal (identified with numbers from 1 to 20), in duplicate. Samples were put into sterile, DNase free, RNase free, cryovials, and stored at −80 °C until further use. Animal handling and sampling was carried out in the frame of the regular veterinary diagnostic procedures of the centre as authorized by the Ministry of Environment and Protection of Land and Sea (Protocol n.0024471/PNM 22/11/2016).

The first set of samples (composed of n. 66 swabs, including the duplicates of the samples 1b-1c, 2b-2c, 3b-3c) was shipped in dry ice in July 2017 to the Centre for Interdisciplinary Research in Animal Health (CIISA, University of Lisbon, Portugal), where the samples were diluted in 600 μL of Hank’s Balanced Salt Solution (Thermo Scientific, Waltham, MA, USA), incubated in a dry bath at 37 °C for 10 min and then stored at −80 °C, until nucleic acid extraction.

The second set of samples (composed of n. 54 duplicate samples but not including the duplicates of the samples 1b-1c, 2b-2c, 3b-3c, as explained above) was shipped in dry ice in December 2018 to the National Reference Laboratory for Animal Chlamydioses, Istituto Zooprofilattico Sperimentale della Lombardia e dell’Emilia Romagna, (IZSLER, Pavia, Italy), where the samples were stored at −80 °C, until nucleic acid extraction.

### 2.2. Chlamydiaceae Screening

At CIISA, the samples were processed for total DNA extraction using a DNeasy Blood & Tissue Kit (Qiagen, Hilden, Germany) following the manufacturer’s instructions, and eluted in a final volume of 60 μL. Total DNA quantification and purity was determined using a NanoDrop 2000C Spectrophotometer (Thermo Scientific, Waltham, MA, USA). The extracted DNA was stored at −80 °C until further use.

The detection of *Chlamydiaceae* DNA was performed by quantitative PCR (qPCR), targeting the 23S rRNA gene, according to a previously described method [36] with minor modifications. A previous positive sample for *C. felis* was used as a positive control of the PCR reaction, and negative controls were also included. Amplification reactions were performed using 5 μL of template DNA in a total volume of 12.5 μL containing: 6.25 μL of SensiFAST™ Probe Hi-ROX Kit (Bioline, London, UK), 0.314 μL of MilliQ water, 0.312 μL of each primer (i.e., TQF, TQR, provided by Stabvida, Caparica, Portugal) at 36 μM, and 0.312 μL of TaqManR probe (labelled FAM/TAMRA, Applied Biosystems, Waltham, MA, USA) at 10 μM. Amplification conditions were: incubation at 94 °C for 10 min, followed by 40 cycles of 95 °C for 15 s and 60 °C for 1 min. Amplification reactions were performed in a StepOnePlus thermal cycler (Applied Biosystems, Waltham, MA, USA).

We selected six samples for additional analyses owing to time and resource constraints, choosing the two samples with the lowest threshold cycle from the qPCR (6a and 8a) and adding the other samples of the corresponding sea turtles (6a-6b-6c and 8a-8b-8c). These samples were amplified by conventional PCR, targeting the 23S rRNA signature sequence of all Chlamydiales, using a previously described method [36], with minor modifications. Amplification reactions were performed using 2.5 μL of the qPCR products in a total volume of 25 μL containing: 15 μL of 5 Prime Master Mix (5 Prime, Hamburg, Germany), 5.5 μL of MilliQ water, 1 μL of each primer (i.e., U23F, 23SIGR, provided by Stabvida, Caparica, Portugal) at 10 μM. Amplification conditions were: incubation at 94 °C for 2 min, followed by 40 cycles of denaturation at 94 °C for 30 s, annealing at 51.5 ± 7 °C for 30 s, and strand extension at 68 °C for 30 s. After cycling, the reaction mixtures were incubated at 68 °C for 10 min and then were held at 4 °C. Amplification reactions were performed in a Doppio thermal cycler (VWR). The obtained amplicons were analysed in a 1.5% agarose gel stained with 0.05 μL/mL of GelRed (Biotium, Fremont, CA, USA) in 1× Tris-Acetate-EDTA, and visualized by ChemiDocTM XRS+ System (Bio-Rad, Hercules, CA, USA). Visible bands were purified using Qiaex II Gel Extraction Kit (Qiagen, Hilden, Germany), quantified in a NanoDrop 2000C Spectrophotometer (Thermo Scientific, Waltham, MA, USA), and cloned into plasmid vectors with the Clone JET PCR Cloning Kit (Thermo Scientific, Waltham, MA, USA), according to the manufacturers’ instructions. The amplicons were subsequently sent for sequencing by Sanger sequencing at Stabvida (Caparica, Portugal) and the specificity of the nucleotide sequences was compared through Blast analysis at http://blast.ncbi.nlm.nih.gov/Blast.cgi (accessed on 19 January 2022) with Chlamydiales sequences available in the GenBank. 

Three oropharyngeal swab samples (18a, 19a, 20a) were further analysed in order to detect *Chlamydiaceae* of potential zoonotic interest. A conventional nested PCR, targeting a partial sequence of the 16S rRNA of three *Chlamydiaceae* species (i.e., *C. trachomatis*; *C. psittaci*; *C. pneumoniae*), was performed as described by Messmer et al. [37], with minor modifications. A previous positive sample for *C. trachomatis* was used as a positive control, and negative controls were also included. The first round of amplification was performed in a total volume of 25 μL, with 5 μL of template DNA, 12.5 μL of Accustart II PCR Supermix (Quantabio, Beverly, MA, USA), 1.25 μL of MilliQ water and 2.5 μL of each primer (provided by Stabvida, Caparica, Portugal) at 20 μM. PCR conditions were: 2 min at 95 °C, followed by 40 cycles of denaturation at 94 °C for 1 min, annealing at 55 °C for 30 s, and strand extension at 72 °C for 1 min. After cycling, the reaction mixtures were incubated at 72 °C for 5 min and then were held at 4 °C. The same PCR conditions were applied to the second round of amplifications, which were performed utilizing 1 μL of the first reaction in a total volume of 25 μL, with 12.5 μL of Accustart II PCR Supermix (Quantabio, Beverly, MA, USA), 6.5 μL of MilliQ water and 2.5 μL of each primer (provided by Stabvida, Caparica, Portugal) at 20 μM. Amplification reactions were performed in a Doppio thermal cycler (VWR, Radnor, PA, USA). The PCR products were analysed in a 1.5% agarose gel stained with 0.05 μL/mL of GelRed (Biotium, Fremont, CA, USA) in 1x Tris-Acetate-EDTA, and visualized by ChemiDocTM XRS+ System (Bio-Rad, Hercules, CA, USA). Visible bands were purified using Qiaex II Gel Extraction Kit (Qiagen, Hilden, Germany), and quantified in a NanoDrop 2000C Spectrophotometer (Thermo Scientific, Waltham, MA, USA). The amplicons were subsequently sent for sequencing by Sanger sequencing at Stabvida (Caparica, Portugal) and the specificity of the nucleotide sequences was compared through Blast analysis at http://blast.ncbi.nlm.nih.gov/Blast.cgi (accessed on 19 January 22) with Chlamydiales sequences available in the GenBank.

### 2.3. Detection of Potential Zoonotic Chlamydiaceae

At IZSLER, DNA was extracted from swabs using a commercial kit, NucleoSpin^®^ Tissue Kit (Macherey-Nagel, Düren, Germany) according to the manufacturer’s instructions and eluted in a final volume of 100 μL. The DNAs were screened for *Chlamydiaceae* by a qPCR targeting 23S rRNA gene [38] using a final concentration of 0.6 μM of each primer and 0.3 μM of probe. For the PCR reaction, the GoTaq^®^ Probe qPCR Master Mix (Promega, Madison, WI, USA) was used with an internal control of amplification (TaqMan^®^ Exogenous Internal Positive Control Reagents, Life Technologies, Carlsbad, CA, USA). Positive (*C. psittaci*) and negative controls were also included in each run. The amplification cycle consisted of 2 min at 95 °C, followed by 45 cycles of denaturation at 95 °C for 15 s, annealing and extension at 60 °C for 1 min. The species of *Chlamydia* (*C. pecorum, C. psittaci* and *C. pneumoniae*) were identified by species-specific qPCR [39,40] using the GoTaq^®^ Probe qPCR Master Mix (Promega, Madison, WI, USA) with a final concentration of 0.6 μM of each primer and 0.25 μM of probe. The amplification cycles were the same used for the screening qPCR.

## 3. Results

### 3.1. Chlamydiaceae Screening

All 66 samples examined at CIISA yielded positive results to the screening for *Chlamydiaceae*, with a mean threshold cycle (Ct) of 32.25 ± 0.25 (ranging from 28.83 to 39.11, as shown in Table 1).

Four out of the six selected samples (6a-6b, 8a-8b) resulted in being positive to further amplification for Chlamydiales (Supplementary Materials Figure S1), and the corresponding purified amplicons were sent for sequencing. We obtained recombinant bacterial colonies only from the sample 6a, which were sent for sequencing (Supplementary Materials Sequences S1). However, Blast analyses resulted in inconclusive identification (Table 2), with all sequences’ closest hits being non-*Chlamydia* species.

None of the three oropharyngeal swab samples resulted in being positive for the amplification reaction for *C. trachomatis*. On the contrary, one oropharyngeal swab sample (18a) yielded amplicons of the expected molecular weight both for *C. psittaci* and *C. pneumoniae* (Supplementary Materials Figure S1). Four purified amplicons, two for each positive reaction, were sent for sequencing (Supplementary Materials Sequences S1), Blast analyses confirmed these to be sequences from *Chlamydia* species but could not conclusively identify a particular *Chlamydia* species (Table 2).

### 3.2. Detection of Potential Zoonotic Chlamydiaceae

Eleven out of the 54 samples, examined at IZSLER, yielded positive results for the screening for *Chlamydiaceae*, specifically three oropharyngeal, six ocular-conjunctival, and two nasal swab samples, belonging to eight turtles (3a, 9b, 10a-10b, 11b, 13b, 15c, 18a-18b-19c, 20b, as shown in Table 3).

None of these eleven samples resulted in being positive for the amplification reaction for *C. psittaci*, nor for *C. pecorum*. On the contrary, two samples, one oropharyngeal and one ocular-conjunctival belonging to the same turtle (18a and 18b), resulted in being positive for the amplification reaction for *C. pneumoniae* (Table 3).

## 4. Discussion

The current study detected the presence of *Chlamydiaceae* from oropharyngeal, ocular-conjunctival, and nasal swabs of clinically healthy loggerhead sea turtles. This is the first instance of chlamydial agents being reported for sea turtles since the outbreak in a green turtle farm in 1994 [33].

The chlamydial host range is wider than previously thought, with more than 400 host species described worldwide, mostly from wildlife. An expanding number of chlamydial species has been detected in wild animals; nevertheless, the most frequently reported ones belong to the *Chlamydiaceae*, likely because of the interest they attract as veterinary and human pathogens (e.g., *C. abortus*, *C. pecorum*, *C. pneumoniae*, *C. psittaci*) [2,10]. Several chlamydial species have been described in reptiles, but *C. psittaci* and *C. pneumoniae* are currently considered the most widespread [9,16,17,21,22,23].

Depending on the host and the chlamydial species involved, the course of infection might range from acute, to chronic, or remain subclinical. Chlamydiae might act as opportunistic pathogens in reptiles, and be detected also in clinically inconspicuous carriers [2,19,24,41]. This is likely our case, given the absence of clinical signs in the examined sea turtles, and the low bacterial load in the swab samples, as suggested by the elevated Ct from the real-time amplification. We presume that our sea turtles were already hosting chlamydial agents at rescue, and the possibility of infection during rehabilitation is diminished because of the maintenance procedures adopted in the centre, including: individual tanks, effective water filtering systems with UV light and ozone and feeding with frozen products intended for human consumption.

Although the health status of our animals should exclude any pathogenic role, it is important to note that *Chlamydiaceae* include well-known pathogens, and the health implications of these agents remain unclear, especially on endangered wild populations [2,10,12]. In addition to the impacts on wildlife health, particular attention should be paid to the role of wild animals in the transmission of chlamydial infections to humans [9,10,11,12,42].

*C. psittaci* is indeed responsible for the most important chlamydial zoonoses, ornithosis/psittacosis, reported to cause disease in humans from influenza-like symptoms to severe pneumonia, with potential complications to other organs (e.g., heart, liver, kidney, brain) [11,12]. Since *C. psittaci* has mainly been considered an avian pathogen, birds have always been regarded as the main route of transmission [43]. However, *C. psittaci* has been detected from other animals, including reptiles, which might have a role in its dissemination [3,17].

*C. pneumoniae* is considered a serious threat to human health, as it is implicated in acute and chronic respiratory infections. Additionally, this agent has been associated with several chronic diseases, such as coronary heart disease, Alzheimer’s disease, multiple sclerosis, reactive arthritis and asthma [2,3,9,15,19,42]. Several animals might serve as a source of infection for *C. pneumoniae*, although the transmission is still a subject of study [10,13,17,19]. Interestingly, the sequences obtained from amphibian and reptile samples showed high similarities with those of human origin, suggesting that *C. pneumoniae* in humans might have derived from these animals [13,16,19,23,44].

Besides these two pathogens, the expanding range of recently discovered chlamydial species, which might represent an additional risk for human and animal health, should be considered [2,14,21]. Indeed, the progress in molecular methods has led to the detection of a number of uncultured chlamydiae, referred to as Chlamydia-like organisms, that still need proper characterization [2,3,35]. Given the scarce information on the pathogenic potential of these agents, their zoonotic implications cannot be excluded, especially considering their phylogenetic similarities to pathogens such as *C. psittaci* and *C. pneumoniae* [21,34]. For example, snakes have been indicated to serve as hosts for novel chlamydial species (*C. serpentis*, *C. poikilothermis*, *Candidati C. corallus* and *C. sanzinia)*, related to *C. pneumoniae* and potentially pathogenic [5,23,34,35].

In the present investigation, it was not possible to identify to the species level the *Chlamydiaceae* detected from our group of sea turtles, although the PCR analyses revealed some positive results for *C. psittaci* and *C. pneumoniae*, because the subsequent nucleotide alignments were not consistent with the identity cut-offs proposed for classification [4,6]. Therefore, we should refer to these isolates as Chlamydia-like organisms. In this respect, the apparent discrepancy between the qPCR findings and the sequencing results in the limited number of further investigated samples could be ascribed to several factors, including PCR method, amplification protocol, and primers targeting different genes at various taxonomic levels. The high sensitivity of qPCR might have allowed the detection of *Chlamydiaceae*, although with the potential amplification of genetically-related organisms, whose taxonomic assignment might be difficult, as indicated by blast analyses. When we used a more selective gene target, shared by microorganisms within the same genus, we were able to obtain a chlamydial sequence. Furthermore, the different results obtained from the two research facilities could be explained by low bacterial loads, low nucleic acid concentration, and the potential DNA degradation during storage (the duplicate set of samples was analysed about one year after the first set).

Although this might represent a study limitation, our results are in line with previous data, reporting the detection, in tortoises and other reptiles, of non-classified chlamydial strains, possibly related to *C. pecorum*, *C. pneumoniae* or *C. psittaci* [5,9,21,22,41]. This might be indicative of microorganisms not belonging to any known chlamydial species, as also previously suggested for some avian species [45,46,47]. More importantly, the extensive presence of chlamydial agents in the examined loggerhead sea turtles highlights the expanded host range of chlamydiae and the role of these reptiles as important reservoirs, requiring further investigations in order to determine the exact taxonomic identity of these microorganisms and to better understand their pathogenic and zoonotic potential.

## 5. Conclusions

Chlamydiae include a wide range of microorganisms of great significance for animal and human health, although our knowledge of many aspects of chlamydial biology is still limited. The current study intended to highlight the importance of chlamydial agents in sea turtles, which have been overlooked for a long time. The detection of chlamydial DNA in the examined loggerhead sea turtles, in the absence of clinical signs, suggests that chlamydiae act as opportunistic pathogens in this species. However, this finding confirms the identification of sea turtles as carriers of these microorganisms, contributing to their dissemination, even if asymptomatic. The possible impacts on sea turtle health and conservation still need to be elucidated, as well as the potential threat posed to public health, especially considering the professional categories working with these reptiles (e.g., wildlife carers, veterinarians, herpetologists). Therefore, further investigations are required to fully characterize the chlamydial agents hosted by sea turtles, in terms of taxonomy, pathogenicity and epidemiology.

## Figures and Tables

**Table 1:** Detection of Chlamydial DNA from oropharyngeal, ocular-conjunctival and nasal swabs of 20 loggerhead sea turtles (performed at CIISA). Threshold cycle (Ct) of the quantitative PCR (qPCR) targeting the *Chlamydiaceae* 23S rRNA gene, and results of the additional amplifications on a sub-set of samples ^1^.

Sample ID	Nucleic Acid (ng/µL)	qPCR (Ct)	Additional Amplifications ^1^
1A	9.7	29.84680	-
1B ^2^	3.3	31.90328	-
1C ^2^	2.1	31.80111	-
2A	8.5	29.61391	
2B ^2^	1.3	39.11171	
2C ^2^	1.9	33.49944	
3A	5.4	32.20633	-
3B ^2^	2.8	35.43770	
3C ^2^	1.9	31.70593	-
4A	3.0	31.98068	-
4B	13.4	34.58099	-
4C	1.3	35.92667	-
5A	5.3	30.92942	-
5B	1.6	34.56563	-
5C	1.8	35.63390	-
6A ^1^	9.2	28.83243	Chlamydiales
6B ^1^	2.1	31.88915	Chlamydiales
6C ^1^	1.7	33.06067	-
7A	7.2	31.16279	-
7B	5.2	30.37660	-
7C	1.1	36.90090	-
8A ^1^	7.3	28.89963	Chlamydiales
8B ^1^	3.0	32.38595	Chlamydiales
8C ^1^	1.4	31.53216	-
9A	2.8	31.58239	-
9B	1.8	33.50734	-
9C	1.1	32.81907	-
10A	3.3	30.94317	-
10B	1.9	33.09943	-
10C	1.3	33.46568	-
11A	5.8	30.21389	-
11B	2.9	33.70123	-
11C	3.1	33.34898	-
12A	3.8	30.17657	-
12B	2.5	33.19109	-
12C	0.9	32.72903	-
13A	4.9	31.20990	-
13B	3.8	30.83905	-
13C	1.2	33.44158	-
14A	3.9	29.35830	-
14B	16.1	31.06517	-
14C	1.3	31.41955	-
15A	4.0	30.92249	-
15B	1.9	32.15538	-
15C	1.0	31.38957	-
16A	5.1	30.77452	-
16B	1.5	31.25544	-
16C	1.5	31.95325	-
17A	5.2	30.14268	-
17B	2.9	32.57115	-
17C	2.4	32.85367	-
18A ^1^	6.4	31.03912	*C. psittaci* and *C. pneumoniae*
18B	2.6	31.28868	-
18C	3.1	30.82862	-
19A ^1^	2.7	35.64708	-
19B	1.7	33.34993	-
19C	1.5	31.18465	-
20A ^1^	4.0	31.96051	-
20B	1.5	32.90759	-
20C	2.5	32.80456	-

^1^ Samples 6a, 6b, 6c, 8a, 8b, 8c were amplified by a conventional PCR targeting the 23S rRNA signature sequence of all Chlamydiales. Samples 18a, 19a, 20a were amplified by a conventional nested PCR, targeting a partial sequence of the 16S rRNA for *C. trachomatis*, *C. psittaci* and *C. pneumoniae*. ^2^ Duplicate samples were not reported in the table.

**Table 2:** Blast analysis results. Top hit from unfiltered blastn suite (NCBI BLAST^®^) for each sequence included in the Supplementary Materials Sequences S1.

Sequence ID	Scientific Name	Query Cover	E Value	Per. Identity	Accession
6b	*Tenacibaculum singaporense*	97%	0.0	93.61	CP032548.1
8b	*Tenacibaculum singaporense*	96%	0.0	95.71	CP032548.1
pJET 6a1	Uncultured bacterium	85%	6.00E-150	86.16	KX158563.1
pJET 6a2	Uncultured bacterium-	84%	6.00E-150	86.16	KX158563.1
pJET 6a3	*Luteolibacter ambystomatis*	89%	2.00E-165	85.14	CP073100.1
pJET 6a4	*Polaribacter* sp. G4M1	90%	0.0	91.51	CP071795.1
pJET 6a5	*Polaribacter* sp. G4M1	93%	0.0	91.51	CP071795.1
18a *C. pneumoniae*	Chlamydiales bacterium V4346-00	88%	3.00E-95	95.93	AY845420.1
18a *C. psittaci*	Chlamydiales bacterium V4346-00	94%	8.00E-48	96.00	AY845420.1

**Table 3:** Detection of Chlamydial DNA from oropharyngeal, ocular-conjunctival and nasal swabs of 20 loggerhead sea turtles (performed at IZSLER). Results of the quantitative PCR (qPCR) targeting the *Chlamydiaceae* 23S gene. Positive samples were further analysed through quantitative PCR to identify potential zoonotic species (i.e., *C. pecorum*, *C. pneumoniae*, and *C. psittaci*).

Sample ID	qPCR	*Chlamydia* spp.
1A	-	-
2A	-	-
3A	Positive	None
4A	-	-
4B	-	-
4C	-	-
5A	-	-
5B	-	-
5C	-	-
6A	-	-
6B	-	-
6C	-	-
7A	-	-
7B	-	-
7C	-	-
8A	-	-
8B	-	-
8C	-	-
9A	-	-
9B	Positive	None
9C	-	-
10A	Positive	None
10B	Positive	None
10C	-	-
11A	-	-
11B	Positive	None
11C	-	-
12A	-	-
12B	-	-
12C	-	-
13A	-	-
13B	Positive	None
13C	-	-
14A	-	-
14B	-	-
14C	-	-
15A	-	-
15B	-	-
15C	Positive	None
16A	-	-
16B	-	-
16C	-	-
17A	-	-
17B	-	-
17C	-	-
18A	Positive	*C. pneumoniae*
18B	Positive	*C. pneumoniae*
18C	Positive	None
19A	-	-
19B	-	-
19C	-	-
20A	-	-
20B	Positive	None
20C	-	-

## Data Availability

The data presented in this study are available within the text and the supplementary materials.

## Supplementary Materials

The following are available online at https://www.mdpi.com/article/10.3390/ani12060715/s1, Figure S1: Gel electrophoresis of the amplification products from the conventional PCR targeting the 23S rRNA signature sequence of Chlamydiales; Sequences S1: sequences obtained from recombinant bacterial colonies and purified amplicons; Figure S1: Gel electrophoresis of the amplification products from the conventional nested PCR targeting the partial sequence of the 16S rRNA signature sequence of three *Chlamydiaceae* species (i.e., *C. pneumoniae*, *C. psittaci* and *C. trachomatis*).
