# Supplementary material for: Detection of Chlamydial DNA from Mediterranean Loggerhead Sea Turtles in Southern Italy
Source: Animals (Basel). 2022 Mar 11;12(6):715. doi: 10.3390/ani12060715 (PMC8944518; doi:10.3390/ani12060715)
Supplement: Supplementary file 1 [file animals-12-00715-s001.zip › Supplementary Material - Figure S1 w Legend.pdf]

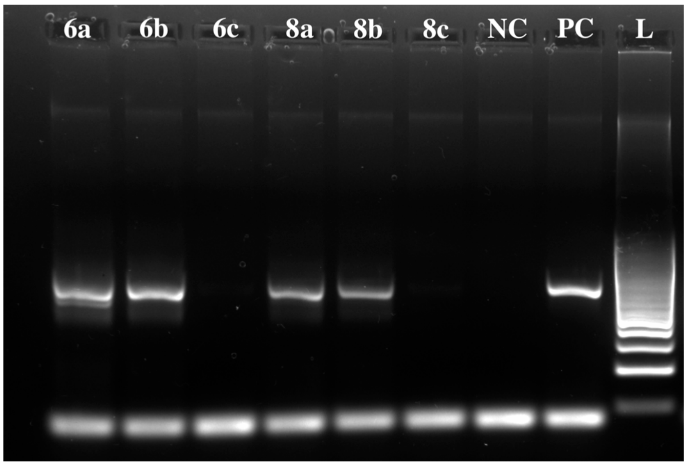

Supplementary Material – Figure S1. Gel electrophoresis of the amplification products from the conventional PCR targeting the 23S rRNA signature sequence of Chlamydiales. A previous *C. felis* positive sample was used as a positive control (PC). 6a-6b-6c = oropharyngeal, ocular-conjunctival and nasal swabs, respectively, from sea turtle 6; 8a-8b-8c = oropharyngeal, ocular-conjunctival and nasal swabs, respectively, from sea turtle 8; NC = Negative control; L = NZYDNA Ladder V (NZYTech).
