# Supplementary material for: Detection of Chlamydial DNA from Mediterranean Loggerhead Sea Turtles in Southern Italy
Source: Animals (Basel). 2022 Mar 11;12(6):715. doi: 10.3390/ani12060715 (PMC8944518; doi:10.3390/ani12060715)
Supplement: Supplementary file 1 [file animals-12-00715-s001.zip › Supplementary Material - Figure S2 w Legend.pdf]

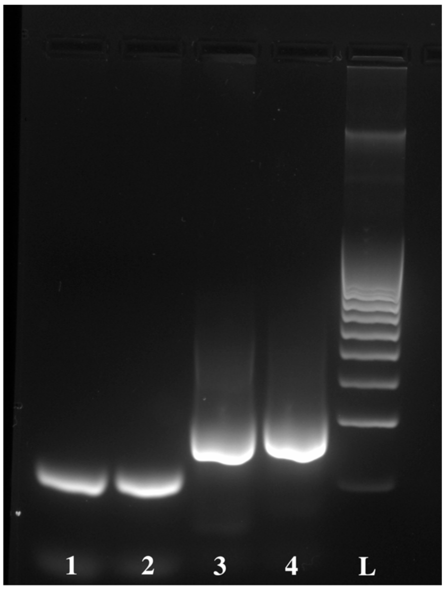


**Supplementary Material – Figure S2.** Gel electrophoresis of the amplification products from the conventional nested PCR targeting the partial sequence of the 16S rRNA signature sequence of three *Chlamydiaceae* species (i.e. *C. pneumoniae*, *C. psittaci* and *C. trachomatis*). 1-2 = Sample 18a (oropharyngeal swab) in duplicate, visible bands of the expected molecular weight for *C. psittaci* (126 bp); 3-4 = Sample 18a in duplicate, visible bands of the expected molecular weight for *C. pneumoniae* (221 bp band); L = NZYDNA Ladder V (NZYTech).
