# Supplementary material for: Detection of Chlamydial DNA from Mediterranean Loggerhead Sea Turtles in Southern Italy
Source: Animals (Basel). 2022 Mar 11;12(6):715. doi: 10.3390/ani12060715 (PMC8944518; doi:10.3390/ani12060715)
Supplement: Supplementary file 1 [file animals-12-00715-s001.zip › Supplementary Material - Sequences S1.pdf]

Supplementary Material – Sequences S1

6a:

Sequence not valid

6b:

CAAAAGGCACGCCGTTACACAGTAAATGTGCTTCGACCGCTTGTAGGCGTACGGTTTC
AGGTTCTCTTTCACTCCCTTACTTAGGGTTCTTTTCACCTTTCCCTCACGGTACTAGTTC
ACTATCGGTCTTTCAGGAGTATTTAGCCTTACCGRATGGTCCCGGTGGATTCATACAG
GATTTCACGTGMCCCGCACTACTCAGGGWACTGCTATCTCTWCTTCGATTACTTATAC
RGGACTATCACCCTCTACGGTTCGTCTTTCCAAACGATTCTAATTCTCTTAGATTCAAA
TATTGCAGCCCTACAACCCCAATATTGCCGTAACAACATTGGTTTGGGCTAATCCGCG
TTCGCTCGCCACTACTAGCGGAATCACTATTGTTTTCTCTTCCTATGGTTACTTARATG
TTTCAGTTCACCACGTTTGCCCCCTTGCGGGTACTATGCCTTCAACATAGTGGGTTGCC
CCATTCGGAAATCTACGGATCATAAAGTATGTGCTTCTCCCCGTAGCTTATCGCAGCT
TATCGCGTCCTTCATCGCCTATCATGCCAAAGGGCA

First unfiltered ten hits from the blastn suite (NCBI BLAST®):

Scientific Name	Query Cover	E value	Per. Identity	Accession
Tenacibaculum singaporense	97%	0.0	93.61	CP032548.1
Tenacibaculum mesophilum	97%	0.0	93.27	CP045192.1
Tenacibaculum mesophilum	97%	0.0	93.27	CP032544.1
Tenacibaculum dicentrarchi	97%	0.0	92.14	CP013671.1
Tenacibaculum todarodis	97%	0.0	91.96	CP018155.1
Tenacibaculum sp. AHE14PA	97%	0.0	91.80	CP058983.1
Polaribacter sp. G4M1	97%	0.0	91.79	CP071795.1
Tenacibaculum sp. AHE15PA	97%	0.0	91.62	CP058982.1
Tenacibaculum jejuense	97%	0.0	91.41	LT899436.1
Tenacibaculum sp. SZ-18	97%	0.0	90.68	CP019335.1

First ten hits filtered by Chlamydiales (taxid:51291)

Scientific Name	Query Cover	E value	Per. Identity	Accession
uncultured Chlamydiales bacterium	30%	1,00E-28	79.89%	KU664237.1

No significant similarity found filtering by Chlamydiaceae (taxid:809)

8a:

Sequence not valid

8b:

GGGGAGGGGCACATATCCTTATGATCCGTAGATTTCCGAATGGGGCAACCCACTATG
TTGAAGGCATAGTACCCGCAAGGGGGCAAACGTGGTGAACTGAAACATCTAAGTAAC
CATAGGAAGAGAAAACAATAGTGATTCCGCTAGTAGTGGCGAGCGAACGCGGATTAG
CCCAAACCAATGTTGTTACGGCAATATTGGGGTTGTAGGGCTGCAACATTAGAATCTA
AGAGAACTAGAATCGTTTGGAAAGACAAACCAAAGAGAGTGATAGTCTCGTATAAGT
AATCGAAGAAGAAATAGCAGTACCCTGAGTAGTGCGGGACACGTGAAATCCTGTATG

AATCCACCGGGACCATCCGGTAAGGCTAAATACTCCTGAAAGACCGATAGTGAACTA
GTACCGTGAGGGAAAGGTGAAAAGAACCCTAAGTAAGGGAGTGAAAGAGAACCTGA
AACCGTACGCCTACAAGCGGTCGAAGCACATTTACTGTGTAACGGCGTGCCTTTTGAG
GAATGGAGCCAATAGCA

First unfiltered ten hits from the blastn suite (NCBI BLAST®):

Scientific Name	Query Cover	E value	Per. Identity	Accession
Tenacibaculum singaporense	96%	0.0	95.71	CP032548.1
Tenacibaculum mesophilum	96%	0.0	94.17	CP045192.1
Tenacibaculum mesophilum	96%	0.0	94.17	CP032544.1
Tenacibaculum dicentrarchi	96%	0.0	94.14	CP013671.1
Tenacibaculum jejuense	96%	0.0	93.55	LT899436.1
Tenacibaculum sp. AHE14PA	96%	0.0	93.39	CP058983.1
Tenacibaculum sp. AHE15PA	96%	0.0	93.19	CP058982.1
Polaribacter sp. G4M1	96%	0.0	92.40	CP071795.1
uncultured bacterium	80%	0.0	96.96	DQ312979.1
Tenacibaculum todarodis	96%	0.0	91.80	CP018155.1

First ten hits filtered by Chlamydiales (taxid:51291)

Scientific Name	Query Cover	E value	Per. Identity	Accession
uncultured Chlamydiales bacterium	32%	4,00E-29	80.46%	KU664237.1

No significant similarity found filtering by Chlamydiaceae (taxid:809)

pJET 6a1:

ATCTTCCGGATGGCTCGAGTTTTTCAGCAAGATGATGCCTTGGCATTGATAGGCGATG
AAGGACGTGGTAAGCTGCGAAAAGCTACGAGGAGGTGCAAACAACCTATGATTCGTA
GATGTCCGAATGGGGAAACCCGCTATATTGAAGATATAGCACCCGCAAGGGAGGAAA
CCCGGGGAACTGAAACATCTAAGTACCCGGAGGAAAAGATAACAATAGTTATTCCGC
AAGTAGTGGCGAGCGAACGCGGAACAGCCCTAAAGTTATATAATTGTTAGTAGAATC
ATTTGGAAAGATGAACCAAAGAGGGTGAAAGTCCCGTAGACGAAAATGGTTATATAA
TGATACGAGTAGGACGGAACCGGTGAAATTCTGTCTGAATATACCAGCACCATCTGG
TAAGGCTAAATACTACTGAGAGACCGATAGCGAACAAGTACTGTGAAGGAAAGGTG
AAAAGAACCCTAAAAAAGGGAGTGAAAAGAACCTGAAACCGTACGCTTACAAGCGG
TCGGAGCCCGTAAGGGTGACGGCGTGCCTTTTGCATGATGAGCCAATCTTTCTAGAAG
ATCTCCTACAATATTCTCAG

First unfiltered ten hits from the blastn suite (NCBI BLAST®):

Scientific Name	Query Cover	E value	Per. Identity	Accession
uncultured bacterium	85%	6,00E-150	86.16	KX158563.1
uncultured Sphingobacteriia bacterium	85%	6,00E-150	86.16	FQ032814.1
uncultured Sphingobacteriia bacterium	85%	6,00E-150	86.27	FQ032813.1
uncultured Sphingobacteriia bacterium	85%	6,00E-150	86.16	FQ032812.1
uncultured Sphingobacteriia bacterium	85%	6,00E-150	86.16	FQ032810.1

uncultured bacterium	65%	2,00E-120	87.53	DQ312751.1
Riemerella anatipestifer	88%	4,00E-117	82.00	CP011859.1
Riemerella anatipestifer RA-YM	88%	4,00E-117	82.00	CP079205.1
Riemerella anatipestifer RA-YM	88%	4,00E-117	82.00	FJ031241.1
Elizabethkingia anophelis	88%	5,00E-116	81.85	CP034594.1

First ten hits filtered by Chlamydiales (taxid:51291) or by Chlamydiaceae (taxid:809)

Scientific Name	Query Cover	E value	Per. Identity	Accession
Chlamydia pneumoniae	42%	1,00E-33	77.86	U68426.2
uncultured Chlamydia sp.	46%	5,00E-33	78.17	MN096564.1
uncultured Chlamydia sp.	40%	5,00E-33	78.17	MN096563.1
uncultured Chlamydia sp.	40%	5,00E-33	78.17	MN096562.1
uncultured Chlamydia sp.	40%	5,00E-33	78.17	MN096559.1
Chlamydia trachomatis	35%	5,00E-33	79.82	U68440.1
Chlamydia muridarum	26%	2,00E-32	83.24	CP042790.1
Chlamydia muridarum	26%	2,00E-32	83.24	CP042777.1
Chlamydia trachomatis	26%	2,00E-32	83.24	CP042803.1
Chlamydia trachomatis	26%	2,00E-32	83.24	CP042802.1

pJET 6a2:

CTGAGAATATTGTAGGAGATCTTCTAGAAAGATTGGCTCATCATGCAAAAGGCACGC
CGTCACCCTTACGGGCTCCGACCGCTTGTAAGCGTACGGTTTCAGGTTCTTTTCACTCC
CTTTTTTAGGGTTCTTTTCACCTTTCCTTCACAGTACTTGTTCGCTATCGGTCTCTCAGT
AGTATTTAGCCTTACCAGATGGTGCTGGTATATTCAGACAGAATTTCACCGGTTCCGT
CCTACTCGTATCATTATATAACCATTTTCGTCTACGGGACTTTCACCCTCTTTGGTTCA
TCTTTCCAAATGATTCTACTAACAATTATATAACTTTAGGGCTGTTCCGCGTTCGCTCG
CCACTACTTGCGGAATAACTATTGTTATCTTTTCCTCCGGGTACTTAGATGTTTCAGTT
CCCCGGGTTTCCTCCCTTGCGGGTGCTATATCTTCAATATAGCGGGTTTCCCCATTCGG
ACATCTACGAATCATAGGTTGTTTGCACCTCCTCGTAGCTTTTCGCAGCTTACCACGTC
CTTCATCGCCTATCAATGCCAAAGCATCATCTTGCTAAAAAACTCGAGCCATCCGGAA
GATCTGGCG

First unfiltered ten hits from the blastn suite (NCBI BLAST®):

Scientific Name	Query Cover	E value	Per. Identity	Accession
uncultured bacterium	84%	6,00E-150	86.16	KX158563.1
uncultured Sphingobacteriia bacterium	84%	6,00E-150	86.16	FQ032814.1
uncultured Sphingobacteriia bacterium	84%	6,00E-150	86.27	FQ032813.1
uncultured Sphingobacteriia bacterium	84%	6,00E-150	86.16	FQ032812.1
uncultured Sphingobacteriia bacterium	84%	6,00E-150	86.16	FQ032810.1
uncultured bacterium	65%	2,00E-120	87.53	DQ312751.1
Riemerella anatipestifer	87%	2,00E-115	81.82	CP011859.1
Riemerella anatipestifer RA-YM	87%	2,00E-115	81.82	CP079205.1

Riemerella anatipestifer RA-YM	87%	2,00E-115	81.82	FJ031241.1
Elizabethkingia anophelis	87%	2,00E-114	81.67	CP034594.1

First ten hits filtered by Chlamydiales (taxid:51291) or by Chlamydiaceae (taxid:809)

**Scientific Name**	**Query Cover**	**E value**	**Per. Identity**	**Accession**
Chlamydia pneumoniae	41%	1,00E-33	77.86	U68426.2
uncultured Chlamydia sp.	45%	5,00E-33	78.17	MN096564.1
uncultured Chlamydia sp.	40%	5,00E-33	78.17	MN096563.1
uncultured Chlamydia sp.	40%	5,00E-33	78.17	MN096562.1
uncultured Chlamydia sp.	40%	5,00E-33	78.17	MN096559.1
Chlamydia trachomatis	34%	5,00E-33	79.82	U68440.1
Chlamydia muridarum	26%	2,00E-32	83.24	CP042790.1
Chlamydia muridarum	26%	2,00E-32	83.24	CP042777.1
Chlamydia trachomatis	26%	2,00E-32	83.24	CP042803.1
Chlamydia trachomatis	26%	2,00E-32	83.24	CP042802.1

pJET 6a3:

CGCCAGATCTTCCGGATGGCTCGAGTTTTCAGCAAGATGATGCCTTGGCATTGATAG
GCGATGAAGGACGTGATAAGCTGCGATAAGCAGCGGTGAGCCGCAAACAGGCTTTGA
CCCGCTGATTTCCGAATGGGGTAACCTGACACCATTAATATGGTGTCGTTCCTTCCTG
AATCAAATAGGGAAGGAGACGCAAGACCCGGTGAAGTGAAACATCTCAGTAACCGG
AGGAAAGGAAAGAGAAATCGATTCCGTGAGTAGTGGCGAGCGAAAACGGAAAAGCC
CGAACCGGGGGATTCATTCCCCGGGGTTGTAGGACCCCGCCGTGGGACCGTCGAGGA
TAGGTGAAGCATTTGGAAAGATGCGACAGAGAGCGTGAGATCCGCGTAGCCGAAATC
CGAGATGGCCCTAGGGAGTTCCTGAGTAGCACGGGACACGTGAAACCCCGTGTGAAT
CTGCGCAGACCACTGCGTAAGGCTAAATACTAACTAGCGACCGATAGTGAACCAGTA
CCGTGAGGGAAAGGTGAAAAGTACCCCTGTGAGGGGAGTGAAATAGAACCTGAAAC
CATGTGCCTACAAGGTGTGGGAGCCCTAGGGTGACCGCGTGCCTTTTGCATGATGAGC
CAATCTTTCTAGAAGATCTCCTACAATATTC

First unfiltered ten hits from the blastn suite (NCBI BLAST®):

**Scientific Name**	**Query Cover**	**E value**	**Per. Identity**	**Accession**
Luteolibacter ambystomatis	89%	2,00E-165	85.14	CP073100.1
Luteolibacter luteus	90%	1,00E-157	84.11	CP051774.1
Sulfuriroseicoccus oceanibius	89%	6,00E-141	82.66	CP066776.1
Opitutae bacterium ISCC 51	89%	3,00E-128	81.33	CP076041.1
Opitutae bacterium ISCC 52	89%	3,00E-128	81.33	CP076040.1
Akkermansiaceae bacterium	66%	6,00E-126	86.14	CP059069.1
uncultured verrucomicrobium HF0070_35E03	89%	6,00E-116	80.27	GU567959.1
uncultured bacterium GRIST08	86%	7,00E-115	80.31	EU795148.1
Opitutae bacterium ISCC 53	89%	1,00E-113	80.07	CP076039.1
uncultured verrucomicrobium MB11C04	89%	7,00E-110	79.74	AY033323.1

First ten hits filtered by Chlamydiales (taxid:51291)

**Scientific Name**	**Query Cover**	**E value**	**Per. Identity**	**Accession**

Uncultured Chlamydiales bacterium	57%	1,00E-40	75.38%	KU664252.1
Uncultured Chlamydiales bacterium	81%	1,00E-39	72.98%	KU664240.1

No significant similarity found filtering by Chlamydiaceae (taxid:809)

pJET 6a4:

TTCCGAATGGCTCGAGTTTTTCAGCAAGATGATGCCTTGGCATTGATAGGCGATGAAG
GACGTGATAAGCTGCGAAAAGCTACGGGGAGGTGCACATAACTTTTGATCCGTAGAT
ATCCGAATGGGGCAACCCGGCATGTTGAAGACATGTCACCGAAAGGAGCAAACCCGG
AGAACTGAAACATCTAAGTACCCGGAGGAAGAGAAAACAATAGTGATTCCGTTAGTA
GTGGCGAGCGAACGCGGATTAGCCCAAACCATATTTGTTACGGCAAATGTGGGGTTG
TAGGACTACGATATTCGATGCTTAATGAATTAGAACAGTTTGGAAAGACTGACCAAA
GAGGGTGATAGTCCCGTATAAGTAAAGAGAGTTATTGATAGTAGTATCCTGAGTAGT
GCGGGGCACGTGTAACCCTGTATGAATCTGTGGGGACCATCCCATAAGGCTAAATAC
TCCTGAAAGACCGATAGTGAACTAGTACCGTGAGGGAAAGGTGAAAAGAACCCTAA
GAAAGGGAGTGAAAGAGATCCTGAAACCGTACGCCTACAAGCGGTCGGAGCCATTCG
TGGTGACGGCGTGCCTTTTGCATGATGAGCCATCTTTCTAGAAGATCTCCTACAATAT
TCTC

First unfiltered ten hits from the blastn suite (NCBI BLAST®):

Scientific Name	Query Cover	E value	Per. Identity	Accession
Polaribacter sp. G4M1	90%	0.0	91.51	CP071795.1
Polaribacter haliotis	90%	0.0	91.38	CP061813.1
Flavobacteriaceae bacterium 10Alg115	90%	0.0	91.11	CP040749.1
Polaribacter sp. SM13	90%	0.0	91.18	CP071869.1
Polaribacter sp. SA4-10	90%	0.0	91.03	CP019331.1
Polaribacter vadi	90%	0.0	91.02	CP017477.1
Polaribacter sp. L12M9	90%	0.0	90.99	CP060695.1
Polaribacter sp. Hel1_33_78	90%	0.0	90.69	LT629794.1
Polaribacter reichenbachii	90%	0.0	90.66	CP019419.1
Polaribacter litorisediminis	90%	0.0	90.53	CP082966.1

First ten hits filtered by Chlamydiales (taxid:51291) or by Chlamydiaceae (taxid:809)

Scientific Name	Query Cover	E value	Per. Identity	Accession
Chlamydia sp. H15-1957-10C	68%	6,00E-48	75.86	LT993738.1
Chlamydia pneumoniae	68%	6,00E-48	75.76	U68426.2
Chlamydia pneumoniae	68%	6,00E-48	75.65	U68422.1
Chlamydia pneumoniae	68%	3,00E-46	75.43	LN849050.1
Chlamydia pneumoniae	68%	3,00E-46	75.43	LN847257.1
Chlamydia pneumoniae	68%	3,00E-46	75.43	LN847255.1
Chlamydia pneumoniae	68%	3,00E-46	75.43	LN847246.1
Chlamydia pneumoniae	68%	3,00E-46	75.43	LN847244.1
Chlamydia pneumoniae	68%	3,00E-46	75.43	LN847241.1
Chlamydia pneumoniae	68%	3,00E-46	75.43	LN847240.1

pJET 6a5:

GGCTCGAGTTTTCAGCAAGATGATGCCTTGGCATTGATAGGCGATGAAGGACGTGA
TAAGCTGCGAAAAGCTACGGGGAGGTGCACATAACTTTTGATCCGTAGATATCCGAA
TGGGGCAACCCGGCATGTTGAAGACATGTCACCGAAAGGAGCAAACCCGGAGAACT
GAAACATCTAAGTACCCGGAGGAAGAGAAAACAATAGTGATTCCGTTAGTAGTGGCG
AGCGAACGCGGATTAGCCCAAACCATATTTGTTACGGCAAATGTGGGGTTGTAGGAC
TACGATATTCGATGCTTAATGAATTAGAACAGTTTGGAAAGACTGACCAAAGAGGGT
GATAGTCCCGTATAAGTAAAGAGAGTTATTGATAGTAGTATCCTGAGTAGTGCGGGG
CACGTGTAACCCTGTATGAATCTGTGGGGACCATCCCATAAGGCTAAATACTCCTGAA
AGACCGATAGTGAACTAGTACCGTGAGGGAAAGGTGAAAAGAACCCTAAGAAAGGG
AGTGAAAGAGATCCTGAAACCGTACGCCTACAAGCGGTCGGAGCCATTCGTGGTGAC
GGCGTGCCTTTTGCATGATGAGCCATCTTTCTAGAAGATCTC

First unfiltered ten hits from the blastn suite (NCBI BLAST®):

Scientific Name	Query Cover	E value	Per. Identity	Accession
Polaribacter sp. G4M1	93%	0.0	91.51	CP071795.1
Polaribacter haliotis	93%	0.0	91.38	CP061813.1
Flavobacteriaceae bacterium 10Alg115	93%	0.0	91.11	CP040749.1
Polaribacter sp. SM13	93%	0.0	91.18	CP071869.1
Polaribacter sp. SA4-10	93%	0.0	91.03	CP019331.1
Polaribacter vadi	93%	0.0	91.02	CP017477.1
Polaribacter sp. L12M9	93%	0.0	90.99	CP060695.1
Polaribacter sp. Hel1_33_78	93%	0.0	90.69	LT629794.1
Polaribacter reichenbachii	93%	0.0	90.66	CP019419.1
Polaribacter litorisediminis	93%	0.0	90.53	CP082966.1

First ten hits filtered by Chlamydiales (taxid:51291) or by Chlamydiaceae (taxid:809)

Scientific Name	Query Cover	E value	Per. Identity	Accession
Chlamydia sp. H15-1957-10C	71%	5,00E-48	75.86	LT993738.1
Chlamydia pneumoniae	71%	5,00E-48	75.76	U68426.2
Chlamydia pneumoniae	71%	5,00E-48	75.65	U68422.1
Chlamydia pneumoniae	71%	2,00E-46	75.43	LN849050.1
Chlamydia pneumoniae	71%	2,00E-46	75.43	LN847257.1
Chlamydia pneumoniae	71%	2,00E-46	75.43	LN847255.1
Chlamydia pneumoniae	71%	2,00E-46	75.43	LN847246.1
Chlamydia pneumoniae	71%	2,00E-46	75.43	LN847244.1
Chlamydia pneumoniae	71%	2,00E-46	75.43	LN847241.1
Chlamydia pneumoniae	71%	2,00E-46	75.43	LN847240.1

18a, *C. pneumoniae*:

TAATGACTTCGGTTGTTATTTAGTGGCGGAAGGGTTAGTAATACATAGATAATTTATT
CTTAACTTGGGAATAACGACTGGAAACGGTCGCTAATACCGAATGTGGTATGTTTASG
CATCTAAATTATATTAAAGAAGGGGATCTTTGGACCTTTCGGTTGTGAGAAAGTCTAT
GGGATATCAGCTTGTTGGTGGGGTAAAAGCCCACCAAGGCGATGACGAGCCCCCCCA
TGCTACGACGAATCCCTTT

First unfiltered ten hits from the blastn suite (NCBI BLAST®):

Scientific Name	Query Cover	E value	Per. Identity	Accession

Chlamydiales bacterium V4346-00	88%	3,00E-95	95.93	AY845420.1
Chlamydiales bacterium V1242-01	88%	3,00E-90	94.57	AY845424.1
uncultured Chlamydia sp.	85%	1,00E-89	95.33	KU669282.1
uncultured Chlamydia sp.	82%	1,00E-88	96.12	KU669286.1
uncultured Chlamydia sp.	82%	1,00E-88	96.12	KU669285.1
uncultured Chlamydia sp.	84%	1,00E-88	95.28	KU669283.1
uncultured Chlamydia sp.	84%	4,00E-88	95.26	KU669281.1
uncultured Chlamydia sp.	82%	6,00E-87	95.63	KU669284.1
Chlamydia sp. 2742-308	88%	6,00E-87	93.67	CP014639.1
uncultured Chlamydia sp.	88%	3,00E-85	93.21	KY928268.1

First ten hits filtered by Chlamydiales (taxid:51291)

Scientific Name	Query Cover	E value	Per. Identity	Accession
Chlamydiales bacterium V4346-00	88%	3,00E-95	95.93	AY845420.1
Chlamydiales bacterium V1242-01	88%	3,00E-90	94.57	AY845424.1
uncultured Chlamydia sp.	85%	1,00E-89	95.33	KU669282.1
uncultured Chlamydia sp.	82%	1,00E-88	96.12	KU669286.1
uncultured Chlamydia sp.	82%	1,00E-88	96.12	KU669285.1
uncultured Chlamydia sp.	84%	1,00E-88	95.28	KU669283.1
uncultured Chlamydia sp.	84%	4,00E-88	95.26	KU669281.1
uncultured Chlamydia sp.	82%	6,00E-87	95.63	KU669284.1
Chlamydia sp. 2742-308	88%	6,00E-87	93.67	CP014639.1
uncultured Chlamydia sp.	88%	3,00E-85	93.21	KY928268.1

First ten hits filtered by Chlamydiaceae (taxid:809)

Scientific Name	Query Cover	E value	Per. Identity	Accession
uncultured Chlamydia sp.	85%	1,00E-89	95.33	KU669282.1
uncultured Chlamydia sp.	82%	1,00E-88	96.12	KU669286.1
uncultured Chlamydia sp.	82%	1,00E-88	96.12	KU669285.1
uncultured Chlamydia sp.	84%	1,00E-88	95.28	KU669283.1
uncultured Chlamydia sp.	84%	4,00E-88	95.26	KU669281.1
uncultured Chlamydia sp.	82%	6,00E-87	95.63	KU669284.1
Chlamydia sp. 2742-308	88%	6,00E-87	93.67	CP014639.1
uncultured Chlamydia sp.	88%	3,00E-85	93.21	KY928268.1
uncultured Chlamydia sp.	88%	3,00E-85	93.21	KY928267.1
uncultured Chlamydia sp.	88%	3,00E-85	93.21	KY928266.1

18a, *C. psittaci*:

GTATAATGACTTCGGTTGTTATTTAGTGGCGGAAGGGTTAGTAATACATAGATAATTT
ATCTTAACTTCGGAATAACGACTGGAAACGGTCGCTATACCGAATGTGGTATGTTTAG
GCATCTAAAACAAC

First unfiltered ten hits from the blastn suite (NCBI BLAST®):

Scientific Name	Query Cover	E value	Per. Identity	Accession
Chlamydiales bacterium	94%	8,00E-48	96.00	AY845420.1

V4346-00				
uncultured Chlamydia sp.	94%	2,00E-44	94.40	KY928268.1
uncultured Chlamydia sp.	94%	2,00E-44	94.40	KY928267.1
uncultured Chlamydia sp.	94%	2,00E-44	94.40	KY928266.1
uncultured Chlamydia sp.	94%	2,00E-44	94.40	KY928264.1
uncultured Chlamydia sp.	94%	8,00E-43	93.60	KU669282.1
uncultured Chlamydia sp.	94%	8,00E-43	93.60	KU669281.1
Chlamydia sp. 2742-308	94%	8,00E-43	93.60	CP014639.1
Chlamydiales bacterium V1242-01	94%	8,00E-43	93.60	AY845424.1
uncultured Chlamydia sp.	98%	3,00E-42	92.31	MT581448.1

First ten hits filtered by Chlamydiales (taxid:51291)

Scientific Name	Query Cover	E value	Per. Identity	Accession
Chlamydiales bacterium V4346-00	94%	8,00E-48	96.00	AY845420.1
uncultured Chlamydia sp.	94%	2,00E-44	94.40	KY928268.1
uncultured Chlamydia sp.	94%	2,00E-44	94.40	KY928267.1
uncultured Chlamydia sp.	94%	2,00E-44	94.40	KY928266.1
uncultured Chlamydia sp.	94%	2,00E-44	94.40	KY928264.1
uncultured Chlamydia sp.	94%	8,00E-43	93.60	KU669282.1
uncultured Chlamydia sp.	94%	8,00E-43	93.60	KU669281.1
Chlamydia sp. 2742-308	94%	8,00E-43	93.60	CP014639.1
Chlamydiales bacterium V1242-01	94%	8,00E-43	93.60	AY845424.1
uncultured Chlamydia sp.	98%	3,00E-42	92.31	MT581448.1

First ten hits filtered by Chlamydiaceae (taxid:809)

Scientific Name	Query Cover	E value	Per. Identity	Accession
uncultured Chlamydia sp.	94%	2,00E-44	94.40	KY928268.1
uncultured Chlamydia sp.	94%	2,00E-44	94.40	KY928267.1
uncultured Chlamydia sp.	94%	2,00E-44	94.40	KY928266.1
uncultured Chlamydia sp.	94%	2,00E-44	94.40	KY928264.1
uncultured Chlamydia sp.	94%	8,00E-43	93.60	KU669282.1
uncultured Chlamydia sp.	94%	8,00E-43	93.60	KU669281.1
Chlamydia sp. 2742-308	94%	8,00E-43	93.60	CP014639.1
uncultured Chlamydia sp.	98%	3,00E-42	92.31	MT581448.1
uncultured Chlamydia sp.	98%	3,00E-42	92.31	MN865876.1
uncultured Chlamydia sp.	96%	4,00E-41	92.19	MT581450.1
